# Normothermic Ex Vivo Machine Perfusion for Liver Grafts Recovered from Donors after Circulatory Death: A Systematic Review and Meta-Analysis

**DOI:** 10.1155/2018/6867986

**Published:** 2018-04-23

**Authors:** Jordan J. Nostedt, Daniel T. Skubleny, A. M. James Shapiro, Sandra Campbell, Darren H. Freed, David L. Bigam

**Affiliations:** ^1^Department of Surgery, Division of General Surgery, University of Alberta Hospital, 2D4.41 W.M.C, 8440-112 St., Edmonton, AB, Canada T6G 2B7; ^2^John W. Scott Health Sciences Library, University of Alberta, 2K3.28 W.M.C, 8440-112 St., Edmonton, AB, Canada T6G 2B7; ^3^Department of Physiology, University of Alberta, 7-55 Medical Sciences Building, Edmonton, AB, Canada T6G 2H7; ^4^Department of Biomedical Engineering, University of Alberta, 1098 Research Transition Facility, 8308-114 St., Edmonton, AB, Canada T6G 2V2; ^5^Department of Surgery, Division of Cardiac Surgery, University of Alberta and 4A7.056 Mazankowski Alberta Heart Institute, 11220-83 Ave, Edmonton, AB, Canada T6G 2B7

## Abstract

As a result of donation after circulatory death liver grafts' poor tolerance to cold storage, there has been increasing research interest in normothermic machine perfusion. This study aims to systematically review the current literature comparing normothermic perfusion to cold storage in donation after circulatory death liver grafts and complete a meta-analysis of published large animal and human studies. A total of nine porcine studies comparing cold storage to normothermic machine perfusion for donation after circulatory death grafts were included for analysis. There was a significant reduction in AST (mean difference −2291 U/L, CI (−3019, −1563);* P *≤ 0.00001) and ALT (mean difference −175 U/L, CI (−266, −85); *P* = 0.0001), for normothermic perfusion relative to static cold storage, with moderate (*I*^2^ = 61%) and high (*I*^2^ = 96%) heterogeneity, respectively. Total bile production was also significantly higher (mean difference = 174 ml, CI (155, 193); *P* ≤ 0.00001). Further research focusing on standardization, performance of this technology following periods of cold storage, economic implications, and clinical trial data focused on donation after circulatory death grafts will be helpful to advance this technology toward routine clinical utilization for these grafts.

## 1. Introduction

Liver transplant remains the only definitive therapy for end stage liver disease. However the shortage of quality organs remains significant in the United States with 1673 patients dying while on the waitlist and a further 1227 removed, too sick to undergo transplant during 2015 [1]. Due to organ shortage, there has been a rise in the use of extended criteria donors (ECD). These donors include those with significant steatosis, advanced age, and donation after circulatory death (DCD) liver grafts [2].

DCD grafts represent an important source of organs to expand the donor pool. The number of DCD grafts used continues to increase; however there is also a rise in the percentage of DCD grafts recovered but not transplanted [1]. This is a result of these grafts' poor tolerance to static cold storage (SCS) [3], the current standard for organ preservation. DCD grafts are more prone to reperfusion injury and susceptible to ischemic biliary cholangiopathy. As a result, outcomes of DCD transplants have traditionally been marginal showing lower long-term patient and graft survival and increased biliary complications [4]. More recent results show improved graft and patient survival, though ischemic cholangiopathy is still a frequent complication of DCD grafts [5].

Ex vivo perfusion is now being studied as a method of increasing use of DCD grafts. Studies using hypothermic and subnormothermic perfusion have shown promising results in both large animal [6–9] and clinical studies [10]; however, in marginal grafts such as those from DCD, normothermic machine perfusion (NMP) showed superior graft function and preservation of biliary epithelium in animal models [11, 12]. In addition to organ preservation, NMP offers the advantage of being able to assess graft viability during perfusion under physiologic conditions where the graft is metabolically active. It also provides opportunity to deliver and monitor response to therapies in order to resuscitate marginal grafts prior to transplantation. These added benefits have led to growing research interest in NMP for DCD grafts in an effort to expand the organ donor pool. NMP for DCD grafts have been studied primarily in large animal studies where resource allocation only allows for small study subject numbers, and study design is critical to advance this complex technology. Although not used often in animal research, systematic reviews can have an important role for the development of future studies [13]. To our knowledge this is the first systematic review of NMP for DCD liver grafts with a meta-analysis of published data.

The aim of this paper is to systematically review the current literature comparing NMP to SCS in DCD liver grafts in large animal (pig) and human studies. The secondary aim is to complete a meta-analysis of NMP versus SCS livers in published DCD porcine liver perfusions.

## 2. Methods

### 2.1. Search Strategy

Searches were conducted in Ovid MEDLINE, OVID EMBASE, EBSCO CINAHL, WOS, SCOPUS, Proquest Dissertations and Theses, and PROSPERO by an expert librarian (SC) in June 2017 and updated in July 2017. Searches employed both controlled vocabularies (e.g., MeSH, EMTREE) and key words such as (DCD livers) and (ex vivo perfusion or normothermic perfusion). Search strategies were adapted for each database. Search strategies are available in the supporting information (S1). No limits were applied.

All full text, porcine, and human trials comparing NMP to SCS for the preservation of DCD livers were included for analysis. Studies that did not include DCD livers and those that focused only on hypothermic or subnormothermic machine perfusion were excluded.

### 2.2. Selection of Studies

Titles and abstracts from the primary search were reviewed independently by two authors (JN, DS) for studies that met inclusion criteria. When this was not clear from the titles and abstracts, full text articles were reviewed to determine inclusion.

### 2.3. Outcome Measures

Primary outcomes in ex vivo perfusion studies included assessment of alanine aminotransferase (ALT) and aspartate aminotransferase (AST) levels as markers of hepatocellular damage, as well as bile production and lactate clearance as markers of liver function. Secondary outcomes were histological preservation and hemodynamic stability indicated by hepatic arterial flow. Primary outcomes in orthotopic pig liver transplant studies included posttransplant peak AST, bile production, and graft survival. Secondary outcomes included histologic preservation. Where there was missing data for quantitative analysis, this information was requested via email from the publication corresponding authors. We received two responses but no further data for inclusion. Where possible, this data was estimated from published figures using Adobe Acrobat Reader DC software.

### 2.4. Assessment of Bias

Articles were assessed by two authors (JN, DS) using the Systematic Review Centre for Laboratory Animal Experimentation (SYRCLE) risk of bias assessment tool [14].

### 2.5. Statistical Analysis

A trained statistician performed statistical analysis. Outcomes assessed in the meta-analysis included AST, ALT, total bile production, and hepatic artery flow for perfusion studies, as well as peak AST for transplant studies. They are all continuous variables expressed as mean ± standard deviation (SD). The mean difference (MD) was used as a summary measure of efficacy between groups treated by NMP and SCS. When no SD was provided, a pooled SD was estimated as previously described [15]. Meta-analysis was performed using RevMan 5.3 software. Heterogeneity of studies was assessed and the following cut-offs were applied, low (>25%), moderate (>50%), and high (>75%) as described by Higgins et al. [16].

## 3. Results

### 3.1. Search Results

**Figure 1:**
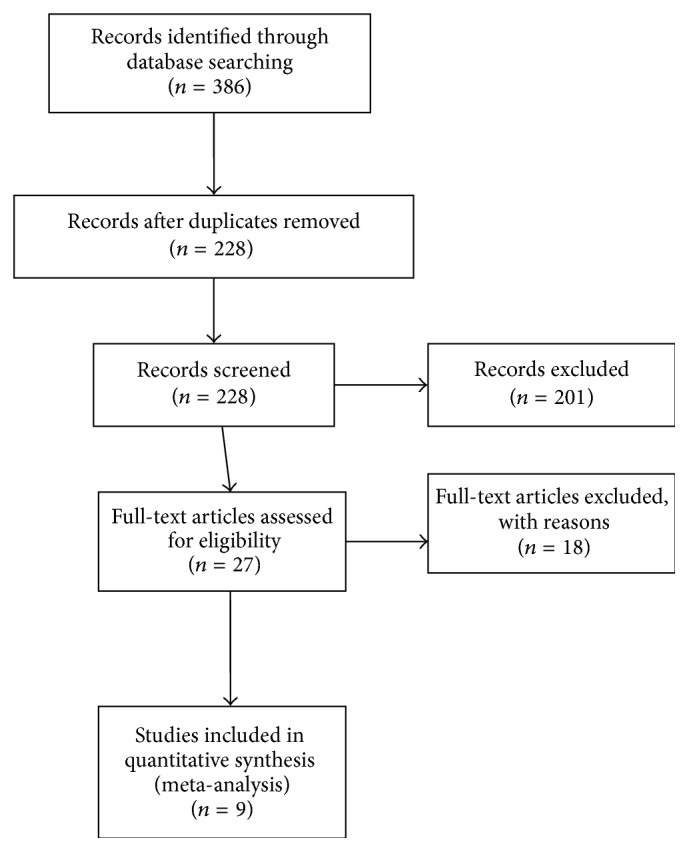
Study selection.

Three hundred and eighty-six titles were identified through our primary search, with 228 remaining for screening after the removal of duplicates. Of these, 201 titles were excluded for the following reasons: published abstract with no complete full text article, comparison of hypothermic or subnormothermic perfusion without NMP, and studies without DCD grafts. Nine articles that directly compared cold storage to NMP for DCD grafts were included for analysis (Figure 1). Six articles were perfusion studies [11, 17–21] and two pig transplant models [22, 23]. One article published results of both a perfusion model and pig transplant model [12]. There are no clinical trials directly comparing SCS to NMP specifically for DCD livers and as such the studies included for analysis were limited to porcine experimental studies. The results of included studies are summarized in Tables 1 and 2.

### 3.2. Pig Liver Ex Vivo Perfusion Studies

**Figure 2:**
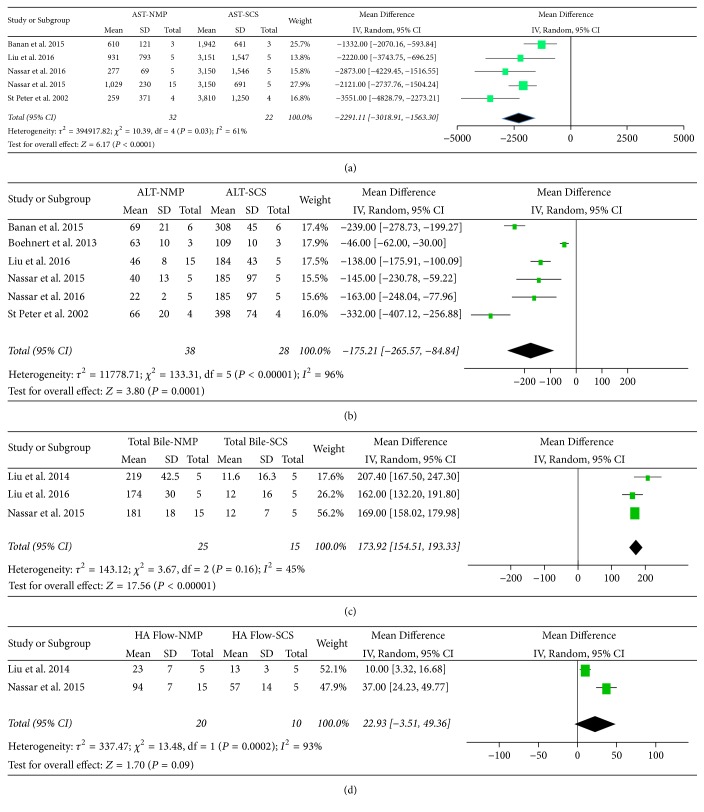
Forest plots showing pooled AST, ALT, and bile production data from porcine liver perfusion studies.

Pooled data showed a significant reduction in AST at the end of the simulated transplant phase in the NMP group relative to SCS (MD = −2291 U/L, CI (−3019, −1563);* P *≤ 0.00001). A similar trend was seen in ALT (MD= −175 U/L, CI (−266, −85); *P* = 0.0001). However the heterogeneity was moderate (*I*^2^ = 61%) and high (*I*^2^ = 90%), respectively, for these two variables (Figure 2).

Total bile production following the simulated transplant phase was significantly higher in the NMP group (MD = 174 ml, CI (155, 193); *P* < 0.00001). There was low heterogeneity (*I*^2^ = 45%) (Figure 2).

There was insufficient data available to perform meta-analysis for lactate clearance.

Limited data was available for hepatic arterial flow. The NMP group did demonstrate higher flows, although this did not reach statistical significance (*P* = 0.09) (Figure 2).

Different histological scoring systems were used by different centers and thus were not suitable for meta-analysis. All perfusion studies showed less necrosis and improved architectural preservation in the NMP group relative to SCS [11, 12, 17–21]. Similarly, NMP demonstrated improved preservation of the biliary epithelium and peribiliary plexus [11, 17, 20].

### 3.3. Pig Liver Orthotopic Transplant Studies

**Figure 3:**
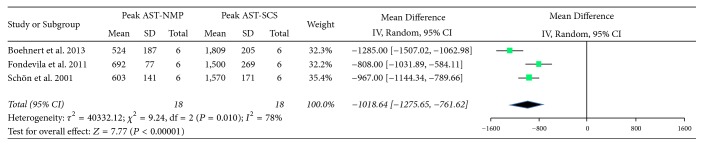
Forest plots showing pooled peak AST data from porcine orthotopic liver transplant studies.

Posttransplant peak AST was lower in the NMP group (MD = −1019, CI (−1276, −762); *P* < 0.00001). There was a high level of heterogeneity (*I*^2^ = 78%) (Figure 3). There was insufficient data available to compare bile production. Graft survival also was not assessed in the meta-analysis, as the recovery period in each of these studies was different (Table 2). Boehnert et al. reported no difference in bile production in the eight hours following transplant after perfusing with acellular solution [12]. Schön et al. showed all grafts transplanted after 60 minutes of WIT and SCS suffered primary graft nonfunction [22]. In an uncontrolled DCD transplant model where normothermic extracorporeal membrane oxygenation was combined with either NMP or SCS, there was 100% five-day survival in the NMP group relative to 83% survival in the SCS group [23].

NMP groups demonstrated less necrosis, sinusoidal swelling, and improved overall architectural preservation relative to SCS groups [22, 23]. One pig transplant model did not report histologic data [24].

### 3.4. Risk of Bias Assessment

The allocation process of animals was unclear in several studies [12, 21–23]; however no other significant sources of bias within the included studies were identified.

## 4. Discussion

The results of this review and meta-analysis must be interpreted with caution, as heterogeneity was high within the perfusion studies limiting the strength of conclusions that can be drawn. Experimental design for the included perfusion studies varied in several fundamental parameters. Major differences included surgical model, duration of preservation and reperfusion, and ex vivo circuit design.

Pigs used as liver donors were 30–40 kg and included landrace [18, 21] and Yorkshire [11, 12, 17, 19, 20], with male pigs used only by Boehnert et al. [25] and gender unspecified ones in one study [21]. The DCD model also varied between studies with the majority inducing cardiac arrest with potassium chloride injection [11, 17–21], while one study induced cardiac arrest via exsanguination [12].

Boehnert et al. work was the only perfusion study to compare SCS to NMP following a period of SCS [12]. The WIT in all included perfusion studies was 60 minutes except for Banan et al. [18] who compared SCS after 40 minutes of WIT to NMP following 20, 40, and 60 minutes of WIT. Following WIT livers were flushed with histidine-tryptophan-ketoglutarate [11, 17–20], University of Wisconsin [12], or Euro Collins [21] cold preservation solutions. Livers were flushed in situ [11, 12, 18, 19, 21] or ex situ [17, 20] with dual perfusion through the hepatic artery and portal vein [11, 17–20] or single arterial flush [21]. One study did not specify if dual vessel flush was used [12].

NMP was then carried out for either 6 [18], 8 [12], 10 [11, 17, 19, 20], or 24 [21] hours. Simulated transplant with whole blood reperfusion was for either 2 [18], 12 [12], or 24 [11, 17, 19–21] hours, which is of important note as transaminase levels were reported at the end of the reperfusion stage.

One study included a dialysis circuit as part of the perfusion setup [18]. Flow was driven by either dual centrifugal pumps [18], the combination of a centrifugal pump and roller pump[11, 17, 19, 20], or a centrifugal pump to perfuse the hepatic artery and the portal vein perfused by gravity [12, 21]. With regard to perfusate used, three studies used whole blood [11, 17, 21], two used dilute whole blood [18, 19], and one used acellular perfusate [12]. The study by Liu et al. is the only one to directly compare different perfusates [20], using Steen solution, Steen solution with washed red blood cells, and whole blood compared to SCS. Hepatocellular injury and liver function were significantly better in the Steen solution with red blood cells and whole blood groups relative to both SCS or Steen solution alone. There was no significant difference between the whole blood or Steen solution with washed red blood cells [20]. Within the included studies there was not enough available data to perform subgroup analysis based on type of perfusate used. However, the results with acellular perfusion [12] to our knowledge have not been replicated and more studies are still needed to determine the optimal NMP perfusate composition for DCD livers; however the results from Liu et al. [20] suggest the need for an oxygen carrier.

In porcine liver transplant models there was also significant study heterogeneity. The posttransplant observation period ranged from eight hours to seven days. Fondevila et al. [23] compared NMP to SCS following a period of normothermic extracorporeal machine oxygenation, which was significantly different from the other included transplant studies. Schön et al. compared SCS to NMP with no period of SCS and all grafts that were exposed to 4 hours of cold storage following 60 minutes of WIT suffered primary nonfunction [22]. This is in keeping with previous data suggesting that even brief periods of cold storage can impact positive effects of NMP [3]. The study by Boehnert et al., however, compared SCS alone to a period of SCS followed by NMP and reported less hepatocellular injury in the NMP group [12], but data from these grafts were only reported for eight hours after transplant and longer-term survival of the grafts was not assessed. In discarded human liver studies, NMP has shown the ability to recover function of damaged livers even after extensive periods of cold storage [26]. Further research to address NMP's ability to safely recover and transplant DCD grafts following periods of cold storage is needed. Devices available for NMP were reviewed by Ravikumar et al. [27] and portable perfusion devices are now available to try and eliminate cold storage time in the transplant sequence for these marginal organs. Whether NMP can successfully recover DCD grafts after periods of cold storage remains an important question that will impact the clinical implementation of ex vivo NMP. The economic impact of these systems has not yet been studied and will also remain a factor in clinical implementation of NMP for DCD grafts. The use of gradual rewarming has shown promise for this population of liver grafts [28–31] and may play an important role moving forward in utilizing machine perfusion after periods of SCS.

NMP has shown capacity to recover function in discarded DCD human liver studies [26, 32–35] and has been used to recover these grafts for clinical transplant [36, 37]. NMP has also been studied as a method to assess which marginal DCD grafts are safely transplantable. A set of viability criteria has been proposed by Mergental et al. [37]. Establishing a standardized set of criteria will be an important goal for clinical implementation of NMP for DCD grafts.

There are phase I clinical trials comparing NMP to SCS [38–40]; however these studies have only limited numbers of DCD and otherwise marginal grafts. To date no randomized control trials have been published comparing NMP to SCS specifically in DCD grafts. Results of a multicenter European randomized control trial (ISRCTN39731134) comparing NMP to SCS, once published, may be pivotal for this technology moving forward into clinical practice.

## 5. Limitations

There was a large amount of heterogeneity amongst the small number of studies as outlined above. These significant differences in experimental design limit the strength of conclusions that could be drawn from meta-analysis. Furthermore, multiple data points included for meta-analysis were estimated from published figures which may differ slightly from the measured values.

## 6. Conclusion

Meta-analysis of published porcine perfusion studies demonstrates that NMP is superior to SCS regarding the preservation of liver architecture and function in DCD grafts. Given significant differences between studies, these results are to be taken with caution. Further study is still required in order to optimize and standardize perfusate composition and to evaluate NMP's role in preservation following periods of cold storage. Clinical studies involving more DCD grafts will help bring this technology closer to clinical implementation. Economic factors need to be considered in subsequent studies to ensure feasibility within current healthcare systems.

## Figures and Tables

**Table 1:** Summary of pig liver perfusion study results.

Perfusion studies	Perfusate	Preservation time (hr)	Simulated transplant phase (hr)	WIT (min)	N-NMP	N- SCS	AST (U/L) NMP	AST (U/L) SCS	ALT (U/L) NMP	ALT (U/L) SCS	NMP total bile (ml)	SCS total bile (ml)	NMP HA flow (ml/min)	SCS HA flow (ml/min)
Boehnert et al. 2013	Steen	4 SCS + 8 NMP vs. 12 SCS	12	60	6	6	__	__	^*∗*^69 ± 21	^*∗*^308 ± 45	__	__	340 ± 85	180 ± 35

Liu et al. 2014	Whole blood	10	24	60	5	5	^*∗*^309	^*∗*^3163 ± 1545	^*∗*^25	^*∗*^186 ± 98	219 ± 42.5	11.6 ± 16.3	23 ± 7 ml/min/100 g liver	13 ± 3 ml/min/100 g

Banan et al. 2015	Saline + whole blood	6	2	40	3	3	610 ± 121	1942 ± 641	63 ± 10	109 ± 10	__	__	^*∗*^504	^*∗*^480 ± 60

Nassar et al. 2015	Acellular solutions + whole blood	10	24	60	15	5	1029 ± 230	3150 ± 691	46 ± 8	184 ± 43	181 ± 18	12 ± 7	94 ± 7 ml/min/100 g	57 ± 14 ml/min/100 g

Liu et al. 2016	Steen + RBC	10	24	60	5	5	^*∗*^931 ± 793	3151 ± 1547	^*∗*^40	185 ± 97	174 ± 30	12 ± 16	__	__

Nassar et al. 2016	Whole blood	10	24	60	5	5	277 ± 69	3150 ± 1546	22 ± 2	185 ± 97	219 ± 43	12 ± 16	__	__

St Peter et al. 2002	Whole blood	24	24	60	4	4	259	3810	^*∗*^66 ± 20	^*∗*^398 ± 74	__	__	1400 ml/min	440 ml/min

*∗*  denotes values estimated from published figures where raw data are not available for analysis. __ denotes data not available for meta-analysis. AST/ALT values are taken at the end of the simulated transplant reperfusion phase. HA flows are ml/min unless units otherwise specified.

**Table 2:** Summary of pig orthotopic liver transplant studies.

Pig transplant studies	Preservation time (hr)	Duration of posttransplant monitoring	NMP *n* =	SCS *n* =	NMP peak AST (U/L)	SCS peak AST(U/L)
Schön et al. 2001	4	7 days	6	6	603 ± 141	1570 ± 171
Fondevila et al. 2011	4	5 days	6	6	692 ± 77	1500 ± 269
Boehnert et al. 2013	12	8 hours	6	6	524 ± 187	1809 ± 205

## Supplementary Materials

Supplementary Materials Supplementary materials include the literature search strategy for this systematic review and meta-analysis (S1).
