# Supplementary material for: Normothermic Ex Vivo Machine Perfusion for Liver Grafts Recovered from Donors after Circulatory Death: A Systematic Review and Meta-Analysis
Source: HPB Surg. 2018 Apr 23;2018:6867986. doi: 10.1155/2018/6867986 (PMC5937385; doi:10.1155/2018/6867986)
Supplement: Supplementary Materials — include the literature search strategy for this systematic review and meta-analysis (S1). [file 6867986.f1.pdf]

S1: Description of literature search strategy

Database: Ovid MEDLINE(R) Epub Ahead of Print, In-Process & Other Non-Indexed Citations, Ovid MEDLINE(R) Daily and Ovid MEDLINE(R) <1946 to Present>
Search Strategy:
----
1 liver transplantation after organ preservation.m_titl. (1)
2 superior preservation of DCD livers.m_titl. (1)
3 criteria for viability assessment of discarded.m_titl. (1)
4 non heart beating donor porcine livers.m_titl. (1)
5 Sanguineous normothermic machine perfusion improves hemodynamics.m_titl. (1)
6 (normothermic machine perfusion and viability testing).m_titl. (1)
7 First human liver transplantation using a marginal allograft.m_titl. (2)
8 ". Impact of Temperature on Porcine Liver Machine Perfusion".m_titl. (1)
9 or/1-8 (9)
10 exp Liver Transplantation/ or exp Liver/ (462813)
11 (liver or livers).mp. [mp=title, abstract, original title, name of substance word, subject heading word, keyword heading word, protocol supplementary concept word, rare disease supplementary concept word, unique identifier, synonyms] (1012778)
12 hepatic.mp. (282677)
13 10 or 11 or 12 (1078000)
14 ((donat* adj2 circulat* death) or (donat* adj2 cardiac* death)).mp. [mp=title, abstract, original title, name of substance word, subject heading word, keyword heading word, protocol supplementary concept word, rare disease supplementary concept word, unique identifier, synonyms] (1178)
15 ("non heart beat*" or NHBD).mp. [mp=title, abstract, original title, name of substance word, subject heading word, keyword heading word, protocol supplementary concept word, rare disease supplementary concept word, unique identifier, synonyms] (1272)
16 dcd.mp. (2013)
17 ((discarded or marginal or declined or rejected) adj5 liver*).mp. [mp=title, abstract, original title, name of substance word, subject heading word, keyword heading word, protocol supplementary concept word, rare disease supplementary

concept word, unique identifier, synonyms] (1048)
18 14 or 15 or 16 or 17 (4764)
19 (exvivo or "ex-vivo" or exsitu or "ex-situ" or excorporeal or extracorporeal or "extra corporeal" or cold storage).mp. [mp=title, abstract, original title, name of substance word, subject heading word, keyword heading word, protocol supplementary concept word, rare disease supplementary concept word, unique identifier, synonyms] (109551)
20 ("normo therm*" or normotherm* or warm perfusion*).mp. [mp=title, abstract, original title, name of substance word, subject heading word, keyword heading word, protocol supplementary concept word, rare disease supplementary concept word, unique identifier, synonyms] (7886)
21 13 and 18 and 19 and 20 (78)
22 exp Rats/ or (rat or rats).mp. (1679155)
23 21 not 22 (60)

Database: Embase <1974 to 2017 July 20>
Search Strategy:
----
1 exp liver transplantation/ or exp liver/ (671770)
2 (liver or livers).mp. [mp=title, abstract, heading word, drug trade name, original title, device manufacturer, drug manufacturer, device trade name, keyword, floating subheading word] (1382551)
3 hepatic.mp. (359985)
4 1 or 2 or 3 (1453549)
5 ((donat* adj2 circulat* death) or (donat* adj2 cardiac* death)).mp. [mp=title, abstract, heading word, drug trade name, original title, device manufacturer, drug manufacturer, device trade name, keyword, floating subheading word] (2430)
6 ("non heart beat*" or NHBD).mp. [mp=title, abstract, heading word, drug trade name, original title, device manufacturer, drug manufacturer, device trade name, keyword, floating subheading word] (1768)
7 dcd.mp. (4169)
8 ((discarded or marginal or declined or rejected) adj5 liver*).mp. [mp=title, abstract, heading word, drug trade name, original title, device manufacturer, drug manufacturer, device trade name, keyword, floating subheading word] (1564)
9 5 or 6 or 7 or 8 (8072)
10 (exvivo or "ex-vivo" or exsitu or "ex-situ" or excorporeal or extracorporeal or "extra corporeal" or cold storage).mp. [mp=title, abstract, heading word, drug trade name, original title, device manufacturer, drug manufacturer, device trade name, keyword, floating subheading word] (162280)
11 ("normo therm*" or normotherm* or warm perfusion* or NMP or "37 adj degree*").mp. [mp=title, abstract, heading

word, drug trade name, original title, device manufacturer, drug manufacturer, device trade name, keyword, floating subheading word] (11768)

12 4 and 9 and 10 and 11 (192)

13 exp rat/ or (rat or rats).mp. (1875464)

14 12 not 13 (159)

15 remove duplicates from 14 (156)
